# *In Vivo* Inhibitory Effect on the Biofilm Formation of *Candida albicans* by Liverwort Derived Riccardin D

**DOI:** 10.1371/journal.pone.0035543

**Published:** 2012-04-24

**Authors:** Yan Li, Yukui Ma, Li Zhang, Feng Guo, Lei Ren, Rui Yang, Ying Li, Hongxiang Lou

**Affiliations:** 1 School of Pharmaceutical Sciences, Shandong University, Jinan, China; 2 Department of Pharmacy, Shandong Provincial Qianfoshan Hospital, Jinan, China; 3 Shandong Pharmaceutical Industry Research Institute, Jinan, China; Institute of Developmental Biology and Cancer Research, France

## Abstract

Riccardin D, a macrocyclic bisbibenzyl isolated from Chinese liverwort *Dumortiera hirsute*, has been proved to have inhibitory effect on biofilms formation of *Candida albicans* in *in vitro* study. Our present study aims to investigate the *in vivo* effect and mechanisms of riccardin D against *C. albicans* biofilms when used alone or in combination with clinical using antifungal agent fluconazole. XTT reduction assay revealed riccardin D had both prophylactic and therapeutic effect against *C. albicans* biofilms formation in a dose-dependent manner when using a central venous catheter related infective animal model. Scanning electron microscope and laser confocal scanning microscope showed that the morphology of biofilms was altered remarkably after riccardin D treatment, especially hypha growth inhibition. To uncover the underlying molecular mechanisms, quantitative real-time RT-PCR was performed to observe the variation of related genes. The downregulation of hypha-specific genes such as *ALS1*, *ALS3*, *ECE1, EFG1, HWP1* and *CDC35* following riccardin D treatment suggested riccardin D inhibited the Ras-cAMP-Efg pathway to retard the hypha formation, then leading to the defect of biofilms maturation. Moreover, riccardin D displayed an increased antifungal activity when administered in combination with fluconazole. Our study provides a potential clinical application to eliminate the biofilms of relevant pathogens.

## Introduction

Biofilms are surface-associated structured microbial communities surrounded by a matrix of extracellular polymeric substances produced by the microorganism cells [1], [2]. Biofilms can form on both natural and artificial surfaces, displaying altered gene phenotypes compared with planktonic cells [3]. Biofilms forming on implanted medical device can cause bloodstream infection, resulting in high rate of morbidity and mortality among hospitalized patients, which has become a major clinical problem [4].

*Candida* species, which also have the ability to develop biofilms on surfaces, represent the fourth most common cause of bloodstream infections in the United States [4]. Of all cases of candidemias, *C. albicans* accounts for up to 63% [5]. Predisposing factors for *Candida* infections include immunosuppressive therapy, broad spectrum antibiotics therapy, HIV infection, diabetes, old age, and use of indwelling devices, such as intravascular catheters [6], [7]. In ICU patients, a total of 72% to 87% of bloodstream infections, including candidemia, are considered to be catheter related [8], [9], with the common mechanism of the fungal cells adherence and biofilms formation on the surfaces of catheters [10]. Infections often result in the removal of the infected catheters in order to eradicate a potential nidus of bloodstream infection [11]. However, catheter removal is not always feasible for patients with coagulopathy or limited vascular access and is associated with increased healthcare expenses as well as complications related to catheter replacement, thus in view of these situations drug treatment is still to be considered [12]. It was proved, however, biofilms-associated cells show resistance against antimicrobials therapy as well as against host defense agents such as phagocytes and antibodies [13]–[16]. For this reason, measures need to be taken to treat with biofilms-associated infection and high levels of drug resistance, and new antifungal agent development is necessary and feasible.

**Figure 1:**
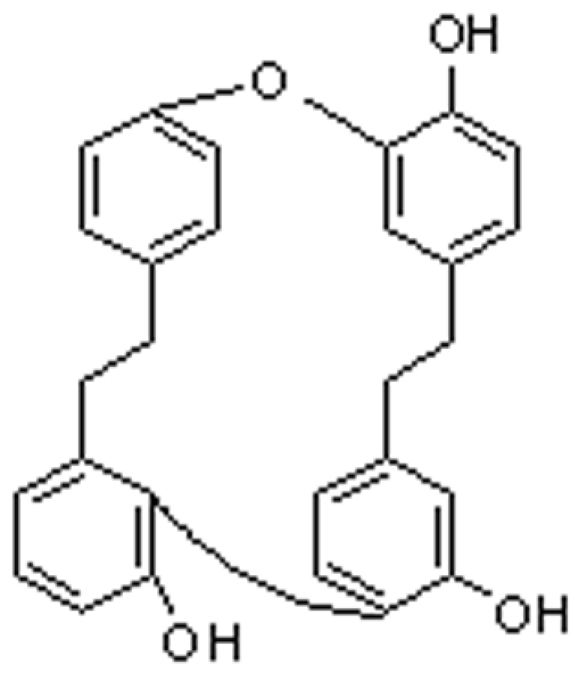
Molecular structure of riccardin D.

Riccardin D (RCD), a macrocyclic bisbibenzyl isolated from Chinese liverwort *Dumortiera hirsute* by our team (Figure 1), has been proved in our previous investigations to have the antifungal activity against *C. albicans* either in planktonic form or in biofilms, with the possible mechanism of hypha formation inhibition [17]–[20]. However, what we have known about RCD is based on *in vitro* studies, and here, we report the efficacy of RCD against *C. albicans* biofilms developed *in vivo* by using a clinically relevant animal model of *C. albicans* biofilms-associated catheter infection. We determined the antifungal efficacy of RCD when administered alone or in combination with traditional antifungal agent fluconazole (FLC), and we also utilized quantitative real-time RT-PCR (qPCR) to investigate the underlying molecular mechanisms of its anti-biofilms effect.

## Methods

### Ethics statement

The use of all the animals (rabbits) in this research was reviewed and approved by Experimental Animals Ethics Committee, Center for Drug Evaluation and Research, Shandong Pharmaceutical Industry Research Institute, Shandong Province, China, under protocol NO. SCXK20080002.

### Organism

*C. albicans* isolate YEM 30 (kindly donated by Dr Michael La Fleur in Northeastern University of USA) was used in this study and stored in medium supplemented with 10% (vol./vol.) glycerol at −80°C. The minimal inhibition concentrations (MICs) of FLC and RCD against this strain in planktonic form were 0.5 µg/ml and 16 µg/ml, respectively, according to the Clinical and Laboratory Standards Institute (CLSI) M27-A_3_ method; while after biofilms formed, the sessile minimal inhibition concentrations (SMICs) were over 64 µg/ml and 128 µg/ml, respectively. Before infection, the strain was grown on YPD solid medium containing 2% agar at 30°C and subcultured in liquid YPD medium (2% tryptone, 1% yeast extract, 2% glucose) in an orbiter shaker with 130 rpm at 30°C.

### Reagent preparation

RCD and FLC were dissolved in the solvent dimethylsulfoxide (DMSO) at the concentration of 20,480 µg/ml as stock solutions and frozen at −20°C. The content of DMSO in the test volume was below 0.5%. The stock solutions were diluted with heparinized saline before use as working solutions. RCD, isolated from liverwort plant *D. hirsute* by our team, has a purity of 98.6%, as determined by high performance liquid chromatography (HPLC). FLC was obtained from the Institute of Biopharmaceuticals of Shandong, China. Propidium iodide (PI) and fluorescein diacetate (FDA) were purchased from Sigma (St Louis, MO, USA).

### Animal models development and biofilms formation

New Zealand White rabbits weighing 2.5 to 3.0 kg were used for all procedures (Animal Centre, Shandong Institute of Pharmaceutical Industry). Animal models were established using a modified method described before [21], [22]. Briefly, an intravenous catheter with a heparin cap (Becton Dickinson Medical Devices Co., Ltd.) was precut by the needle side and sterilized by ethylene oxide. Before the catheter was inserted into the venous, a rectangular polyethylene film (1 cm×2 cm) sterilized by 75% alcohol was curled gently and put into the lumen of the catheter with a stainless steel needle under aseptic condition. Sterilization of the film was verified by colony count. Rabbits were given intravenous anesthesia with pentobarbital sodium (3%, w/v, 40 mg/kg of body weight). At the same time, the animals were given penicillin and gentamycin to prevent the surgical site infection. The right cervical, shoulder, and scapular regions were shaved with an electric clipper and sterilized with 75% alcohol followed by betadine solution. An incision was made in the right anterolateral cervical region, exposing the external jugular vein. A segment of the external jugular vein, just distal to the bifurcation of the internal and external maxillary veins, was freed from subcutaneous fat. The catheter full of sterile heparinized saline was inserted into the vein caudally 3 cm, with the tip placed in the right anterior vena cava. The proximal and distal ligatures were tied, and blood was withdrawn to test catheter patency. The catheter was then flushed with heparinized saline. Passing a hemostat cephalad through a 1.0-cm incision in the intrascapular region to the external jugular vein incision site created a subcutaneous tunnel. The heparin lock device at the end of the catheter was pulled out through the subcutaneous tunnel. The neck incision and the catheter exit were closed by using 3-0 vicryl sutures and sterilized with betadine solution.

Catheters were infected 24 hours after their placement to allow a conditioning period for deposition of host protein on the surface of the polyethylene film. A standard 300 µl of inoculum (the lumen volume of the catheter) consisting of 10^7^ CFU of *C. albicans* YEM 30, 100 U of heparin (Changzhou Qianhong biochemical pharmaceutical Co., LTD, China), and 0.9% sterile normal saline was prepared. The mixture was “locked” in the internal lumen of the catheter and allowed to dwell for a special period of time. Then the inoculum was removed, and replaced with heparinized saline. During the experiment, the catheter was flushed with 300 µl of heparinized saline (100 U) every 24 hours. The animals were observed twice a day for signs of distress or inflammation after the surgery.

### Inhibitory effect of RCD against *C. albicans* biofilms *in vivo*

To determine the prophylactic and therapeutic effect of RCD against *C. albicans* biofilms *in vivo*, we administered RCD solutions to the catheterized animals before and after biofilms formed. The anti-biofilms effect of RCD used in combination with traditional antifungal agent FLC was also studied to evaluate whether the compound had sensitizing effect to the latter.

For the first experiment, RCD were administered once at the early stage of *C. albicans* biofilms development to test the prophylactic effect of RCD against biofilms growth. The animals were catheterized and infected as described above. After 4 hours attachment, the inoculums were removed, and 300 µl of drug solutions containing 100 U of heparin were injected slowly through the heparin caps. The rabbits were randomized into six groups, each consisting of six animals. Group I received 300 µl of heparinized (100 U) saline as the control. Group II, III and IV received 300 µl of RCD solution (at the concentrations of 8 µg/ml, 16 µg/ml and 64 µg/ml, respectively) containing 100 U of heparin. Group V and VI received 300 µl of FLC solution (4 µg/ml), and FLC (4 µg/ml) plus RCD solution (16 µg/ml) containing 100 U of heparin, respectively, to investigate the antifungal effect of RCD against *C. albicans* biofilms when used in combination with FLC. The concentrations of FLC and RCD were based on the result of an *in vitro* study (data not shown). We used FLC with the concentration 8 times higher than the *in vitro* MIC. The drug solutions were locked in the catheter lumens for 8 h, at which time the drug solutions were removed and replaced with heparinized saline. After 24 h from the initiation of infection, animals were sacrificed humanely.

For the second experiment, to assess the therapeutic effect of RCD against pre-existing *C. albicans* biofilms, the drug solutions (at the concentrations of 8 µg/ml, 16 µg/ml and 64 µg/ml, respectively) were injected after 24 hours of *C. albicans* infection. The rabbits were randomized into four groups, each consisting of six animals. Animals received 300 µl of heparinized saline instead of RCD solution as the control. The drug solutions were kept in the catheters for 8 h per day for 5 days consecutively. For daily catheters treatment, RCD lock solutions were replaced by 300 µl of heparinized saline. If the drug solution was difficult to withdraw from the catheter, it could be flushed into the systemic circulation.

After the animals were sacrificed, the catheters were removed under sterile conditions and the polyethylene films were taken out. The films were made smooth gently and flushed with phosphate-buffered saline (PBS, pH 7.4) to remove blood and nonadherent *C. albicans* cells. The specimens were cut into two segments, one for quantitative culture, and the other for confocal laser scanning microscopy examination. Before quantitative culture, the specimens were put into 200 µl sterile PBS, sonicated at 40,000 Hz (Bransonic 1510; Branson Ultrasonics Corp., Danbury, Conn.) for 15 min and then vortexed for 30 s vigorously. The quantitative results were expressed as the mean CFU per milliliter from 6 animals. Based on prior clinical studies, catheters growing 10^2^ CFU or more of *C. albicans* were considered infected [22]. We selected samples randomly for scanning electron microscopy (SEM).

### Quantitative determination of biofilms

For quantitation, after the specimens were flushed with PBS, sonicated and vortexed, the suspension was removed to microtiter plates. We used a 2,3-bis(2-methoxy-4-nitro-5-sulfo-phenyl)-2H-tetrazolium-5-carboxanilide (XTT) reduction assay as previously described to quantify *C. albicans* cells [23]. Briefly, XTT (Sigma) was prepared as a saturated solution at 0.5 mg/ml in Ringer's lactate and filter sterilized and stored at −70°C. Prior to each assay, menadione (10 mM prepared in acetone; Sigma) was added to obtain a final concentration of 1 mM. A 100 µl aliquot of XTT-menadione was then added to each well, and microtiter plates were incubated in the dark for 2 h at 37°C. The colorimetric change (a reflection of the metabolic activity of cells within the biofilms) was measured in a BIO-TEK ELX800 microtiter plate reader at 490 nm. The antifungal effect was measured by comparing the reduction in the mean absorbance of the drug treated wells to that of the drug free control. The experiment was performed in triplicate.

### Catheter biofilms imaging

#### Scanning electron microscopy

Biofilms were assessed after catheters infection with or without drug challenge by SEM observation. Briefly, the infected polyethylene films were flushed with PBS and fixed in 2.5% glutaraldehyde to be prepared for SEM observation. Samples were subsequently washed in distilled water, dehydrated gently by washing in a series of ethanol alcohol (30% for 10 min, 50% for 10 min, 70% for 10 min, 95% for 10 min and 100% for 10 min), and air dried in a desiccator prior to sputter coating with gold. Afterwards, specimens were mounted on the aluminium stubs and coated with gold in a low-pressure atmosphere with an ion sputter coater (Jeol JFC1 100: Jeol, Tokyo, Japan). The topographic features of the biofilms were visualized with a scanning electron microscope (Philips XL30CP) in high vacuum mode at 10 kV and the images were processed for display with Photoshop software (Version 5.0, Adobe Systems Inc., Mountain View, CA).

#### Confocal laser scanning microscopy

The morphology and activity of *C. albicans* biofilms were observed by a confocal laser scanning microscope (Zeiss 710). Before observation, the specimens were stained with FDA and PI as described previously [24], [20]. All metabolically active yeast cells were stained with the FDA dye and visualized with a diffusely distributed green fluorescence, whereas those with damaged membranes were stained with PI and showed fluorescent red. Thus cell viability could be assessed using this dye method. Stained specimens were transferred to a glass slide covered with a coverslip disk for observation. Images were processed for display by using Axiovision 3.x software (Zeiss).

### Quantitative RT-PCR

Quantitative real-time reverse transcription-PCR (RT-PCR) was used to compare mRNA abundances of the genes of interest. *C. albicans* biofilms were formed as described above. RCD solution at the concentration of 64 µg/ml was administered to the catheter after 4 hours attachment and allowed to dwell for 18 hours, while catheters receiving heparinized saline alone worked as the comparison. We did 3–4 animals to obtain enough mRNA for analysis for each experiment. Animals were sacrificed after 48 hours from infection. Total RNA was extracted from the 48-h-old biofilms with or without RCD challenge. The TaqMan MGB probe and primer sets were designed for the target genes *ALS1*, *ALS3*, *ECE1, EFG1, HWP1* and *CDC35*, using Primer Express 1.5 software (Applied Biosystems, Foster City, Calif.). Primer sequences used for amplification of the genes are listed in Table 1. The total RNAs were respectively isolated using the hot phenol method as described elsewhere [24]. Reactions were performed in duplicate according to the kit manufacturer's instructions. We used *ACT1* as the internal control. The experiment was carried out in triplicate. The quantitative data analysis was completed using an Eppendorf Mastercycler Real Time PCR System. The comparative expression value was calculated using the following formula: fold change = 2^−ΔΔCt^.

**Table 1:** Primers used for RT-PCR experiments.

*primer*	*sequence*	*T_m_ (°C)*
***ALS1-F***	5′-ATTGGTAAAGTAACTGTACCA-3′	60.0
***ALS1-R***	5′-CACATTGAATATGCCATGTG-3′	60.0
***ALS3-F***	5′-AGGTTGTTCTTGGGTTTCTG-3′	55.7
***ALS3-R***	5′-TGTAGATGGAGAAGTTG-3′	53.7
***ECE1-F***	5′-GCTGGTATCATTGCTGATAT-3′	60.0
***ECE1-R***	5′-TTCGATGGATTGTTGAACAC-3′	60.0
***EFG1-F***	5′-TATGCCCCAGCAAACAACTG-3′	57.8
***EFG1-R***	5′-TTGTTGTCCTGCTGTCTGTC-3′	57.8
***HWP1-F***	5′-TGGTGCTATTACTATTCCGG-3′	55.75
***HWP1-R***	5′-CAATAATAGCAGCACCGAAG-3′	55.75
***CDC35-F***	5′-TTCATCAGGGGTTATTTCAC-3′	53.7
***CDC35-R***	5′-CTCTATCAACCCGCCATTTC-3′	57.8
***ACT1-F***	5′-AGCTTTGTTCAGACCAGCTGATT-3′	60.0
***ACT1-R***	5′-TTGACCAAACCACTTTCAACTCC-3′	60.0

### Statistical evaluation

Data were described as mean ± SD. The mean CFUs from quantitative catheter cultures were compared by the Student's *t-*test using StatView version 5.0.1 software (SAS Institute, Cary, N.C.). *P*<0.05 was considered significant.

## Results

### Animal models development

**Figure 2:**
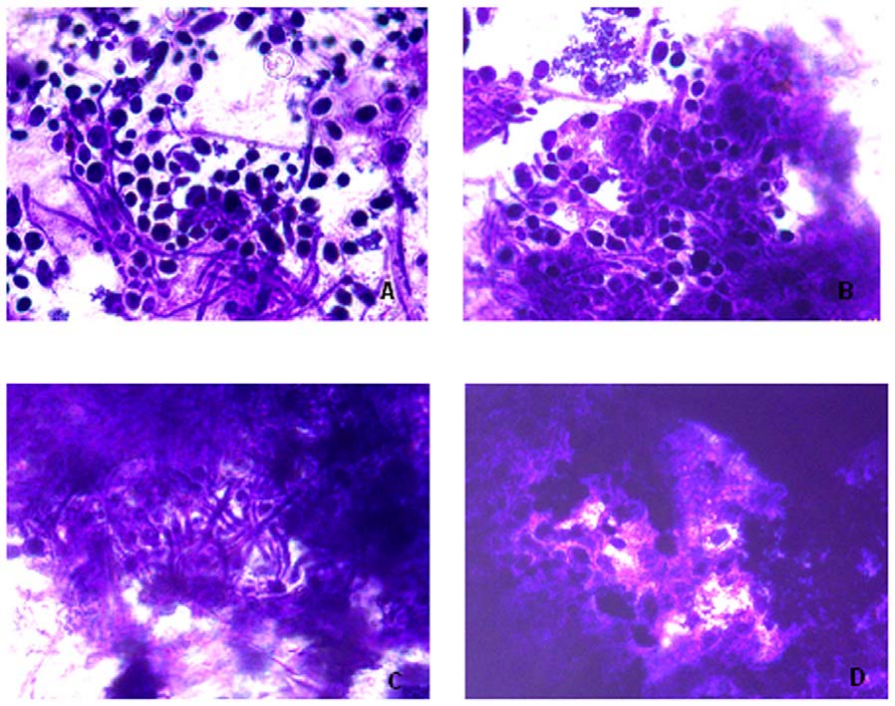
Inverted microscope images of *C. albicans* biofilms formed *in vivo.* *C. albicans* biofilms after 10 h (**A**), 24 h (**B**), 48 h (**C**) and 72 h (**D**) of development (×1,600).

We established central venous catheter (CVC) -associated *C. albicans* biofilms infective rabbit model according to the method described before with slight modification [21], [22]. The catheterized rabbits appeared well during all of the study. After the animals were sacrificed, the polyethylene films in catheters were removed, flushed with PBS, stained by using Gram's method and observed under an inverted microscope (Olympus). Biofilms on the surface were unevenly distributed, with the cells, hypha, and matrix stained blue as shown in Figure 2. In the 10-hour-old biofilms, both yeast and hypha could be observed with a thin layer, and not much matrix. After 24 h development, multiple-layered mature biofilms consisting of an extensive network of yeast cells and hypha embedded in abundant extracellular matrix formed. The biofilms continued to grow and proliferate after 48 h and 72 h. At the late stage of development, the biofilms were embeded with so thick matrix that they could not be observed clearly.

### Efficacy of RCD on *C. albicans* biofilms formed *in vivo*

**Figure 3:**
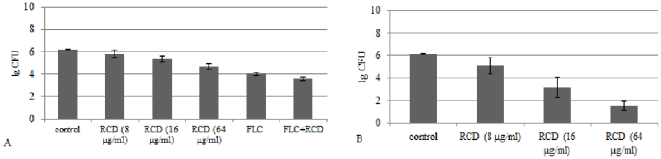
Antifungal effect of riccardin D against *C. albicans* biofilms formed *in vivo*. (**A**) Riccardin D at different concentrations (8 µg/ml, 16 µg/ml and 64 µg/ml), fluconazole (4 µg/ml), or riccardin D (16 µg/ml) combined with fluconazole (4 µg/ml) was administered once to 4-hour-old biofilms and were allowed to dwell in the catheter for 8 h. Animals in control group received heparinized saline instead of drug solution. After 24 h growth of biofilms, the specimens were removed for quantification. (**B**) Riccardin D at different concentrations (8 µg/ml, 16 µg/ml and 64 µg/ml) were administered to the 24-hour-old biofilms, and allowed to dwell for 8 h per day for 5 days consecutively. Animals in control group received heparinized saline instead of drug solution. After treatment of RCD for 5 days, the specimens were removed for colony count.

Using quantitative culture technique, we evaluated the prophylactic efficacy of RCD against *C. albicans* biofilms developed *in vivo* when used alone or combined with FLC, and the therapeutic effect after 5-day-treatment of RCD as well. Results were shown in Figure 3. When administered after 4 hours attachment of *C. albicans* cells, RCD showed an inhibition effect against biofilms growth in a concentration dependent manner (Figure 3 (A)). The cell counts in the control group (catheters were treated with heparinized saline) was (1.5×10^6^±1.4×10^5^) CFU. After the treatment with RCD at the concentrations of 8 µg/ml, 16 µg/ml and 64 µg/ml for 8 h, the cell counts were (7.2×10^5^±3.1×10^5^) CFU, (2.6×10^5^±1.4×10^5^) CFU and (5.6×10^4^±2.8×10^4^) CFU, respectively. The cell counts were significantly decreased in RCD-treated groups at the medium and high concentrations compared with that in the control group (*P* = 0.0276 and *P* = 0.0003, respectively). The quantification of cells treated with FLC alone or in combination with RCD were (1.2×10^4^±4.5×10^3^) CFU and (3.8×10^3^±1.5×10^3^) CFU, respectively (*P* = 0.0004), suggesting a remarkably decrease in *C. albicans* cells and an increased efficacy of drug combination than used alone, which was accordance with the results *in vitro* (data not shown).

For the 5-day-treatment experiment, the control, the low dose and medium dose group each had one catheter clotted, so results were obtained from the unclotted catheters. Against the 24-hour-old biofilms, RCD showed good antifungal effect in a dose-dependent manner. As shown in Figure 3 (B), the results indicated about 4 log_10_, 3 log_10_ and 1.6 log_10_ reduction in biofilms fungal load for the high dose, medium dose and low dose group compared with the control group (*P*<0.0001, *P* = 0.0002 and *P* = 0.0015, respectively). Biofilms were eliminated totally in 3 catheters in high dose group and 2 in medium dose group.

We evaluated the toxicity of RCD by observing animals' reactions and measuring the body weights. The animals showed no obvious adverse reaction or significant loss of body weight during drug use. Similarly, according to our previous work, RCD treatment was generally well tolerated by mice except for slight vein stimulation when administered via tail vein (not published), indicating the safety of RCD. In this study, drug solutions were injected to catheters locally by “antibiotic-lock” technique rather than administered systemically, therefore, systemic and severe drug toxicity to animals could be avoided.

### Biofilms imaging

**Figure 4:**
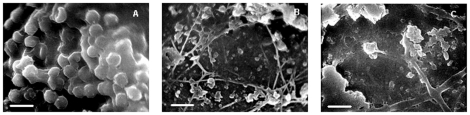
Scanning electron microscopy images of *C. albicans* biofilms formed *in vivo* (×2,000). (**A**) biofilms treated with heparinized saline; (**B**) biofilms after riccardin D treatment for 8 h. The inoculums of YEM 30 were removed after 4 hours growth, and riccardin D solution at the concentration of 64 µg/ml was injected into the catheter and locked in the lumen for 8 h; (**C**) biofilms after 5-day-treatment of riccardin D. The inoculums of YEM 30 were removed after 24 hours growth, and riccardin D solution at the concentration of 64 µg/ml was injected into the catheter and locked in the lumen for 8 h for 5 days consecutively. The bar is 15 µm for the three panels.

Catheters with or without drug challenge were removed from the animals, and the polyethylene films were taken out as the specimens for SEM and confocal laser scanning microscopy images. Figure 4 shows SEM images of *in vivo C. albicans* biofilms with or without RCD (64 µg/ml) treatment. The biofilms in the control group was in large quantity, but in uneven distribution in the polyethylene films. After 24 h of growth *in vivo*, the biofilms consisted of a lot of yeast cells and hypha embedding in much extracellular matrix. We could find some red blood cells not flushed from the biofilms, having similar size with *C. albicans* cells but different phenotype (Figure 4 (A)). For the biofilms treated with RCD at the early stage, however, it was difficult to find biofilms under SEM because of its small amout. In the remaining biofilms, *C. albicans* cells were shrinked, without much hypha or thick extracellular matrix around the cells as the biofilms in the control group (Figure 4 (B)). For the biofilms treated with RCD for 5 days, what could be seen only were some dead cells, fractured hypha and remaining ECM (Figure 4 (C)).

**Figure 5:**
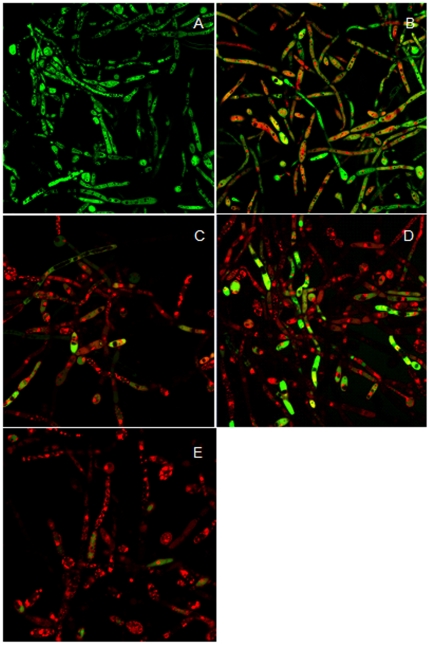
Confocal laser scanning microscopy images of *C. albicans* YEM 30 biofilms developed *in vivo* (×630). After 4 hours of *C. albicans* YEM 30 infection, the inoculums were removed, and riccardin D was administered alone or in combination with fluconazole and allowed to dwell in the catheters for 8 h. The specimens were removed after the animals sacrifice, washed with PBS (pH 7.4) and stained with fluorescein diacetate (FDA) and propidium iodide (PI). Cell viability could be assessed using confocal laser scanning microscopy because healthy cells with an intact membrane were stained fluorescent green, whereas those with damaged membranes stained fluorescent red. (**A**) Biofilms after 24 h of development without drug treatment; (**B**) Biofilms challenged by riccardin D at the concentration of 16 µg/ml; (**C**) Biofilms treated with riccardin D at the concentration of 64 µg/ml; (**D**) Biofilms treated with fluconazole at the concentration of 4 µg/ml; (**E**) Biofilms treated with riccardin D (16 µg/ml) and fluconazole (4 µg/ml) when used in combination.

We also investigated the activity of biofilms cells by staining with FDA and PI, as shown in Figure 5. Images showed that live and dead cells could be distinguished under the confocal laser scanning microscope. For the biofilms without drug treatment, strong green fluorescent was observed because FDA binded to metabolically active cells, suggesting the cells in biofilms were viable (Figure 5 (A)). We found different morphology when biofilms were treated with RCD solutions at the concentrations of 16 µg/ml and 64 µg/ml (Figure 5 (B) and (C)). The cells were stained green or red, with fewer cell counts and hypha compared with the control, indicating the cells in biofilms were inviable because of the damage by the drugs. When using RCD (16 µg/ml) combined with FLC (4 µg/ml), the cells were stained with red color, much smaller and fewer, and with fewer hypha in the visual field (Figure 5 (E)) compared with those challenged by FLC alone (Figure 5 (D)). It could be concluded from these findings that RCD has antifungal efficacy against *C. albicans* biofilms, and there was an increased anti-biofilms effect when RCD and FLC were used in combination.

### Quantitative RT-PCR

**Figure 6:**
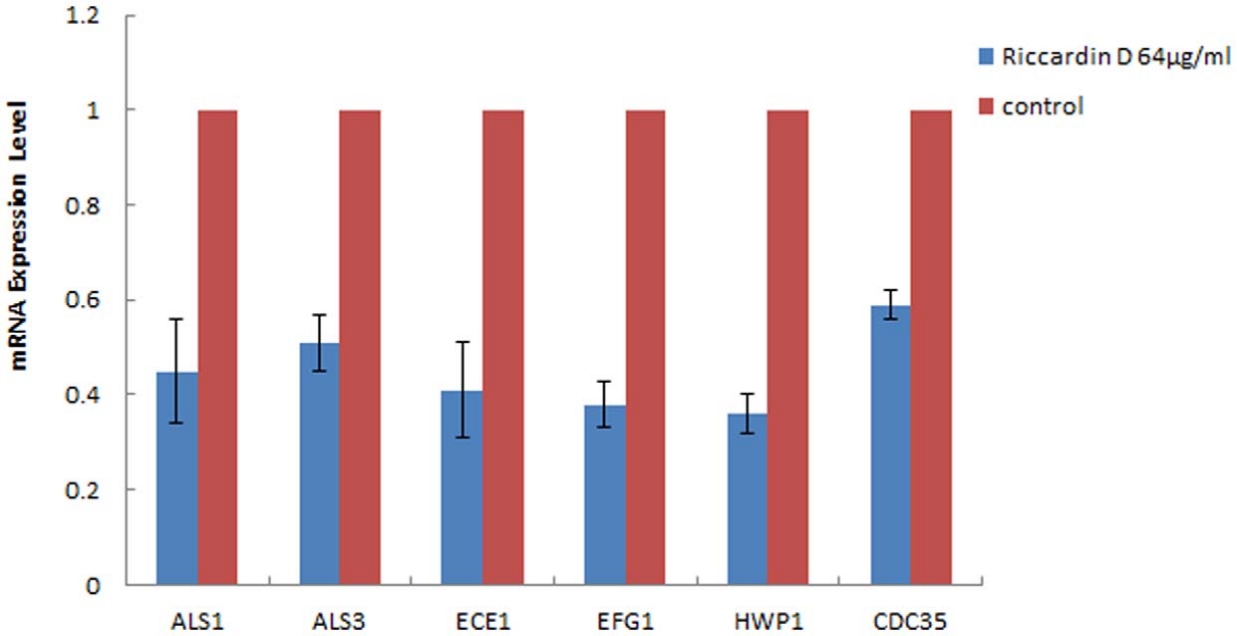
Expression of *C. albicans* biofilms-related genes. Different expression of genes *ALS1, ALS3, ECE1, EFG1, HWP1* and *CDC35* following the treatment with riccardin D (64 µg/ml) for 18 h. The biofilms grew for 48 h totally. *ACT1* works as an internal control.

To elucidate the potential molecular mechanism, we extracted mRNA from cells isolated from the polyethylene films and then utilized qPCR to analyze the expression of hypha-related genes under the treatment of RCD. We did several animals to obtain enough mRNA for analysis, with 14 µg of total *C. albicans* mRNA isolated from the films. The data showed that *C. albicans* hypha-related genes *ALS1, ALS3, ECE1, EFG1, HWP1*, and hypha formation regulator *CDC35,* were all down-regulated following the treatment of RCD at the concentration of 64 µg/ml. The results were shown in Figure 6.

## Discussion

Natural products have been an important source of innovative therapeutic agents in various conditions, including infectious diseases [25]. Our team has been committed to extracting and screening compounds with antifungal activity from liverworts plants. Among these compounds, bisbibenzyls were proved to have a wide range of biological activities such as antifungi, antioxidation, antivirus, antibacteria and cytotoxicity [26]–[29]. In our previous research, bisbibenzyls showed antifungal effects through stimulating the synthesis of farnesol via upregulation of Dpp3, suggesting a potential antifungal application of this kind of compounds [30]. The macrocyclic bisbibenzyl RCD, extracted from Chinese liverwort *Dumortiera hirsute*, structurally consisted of two biphenyl bonds linked by diaryl ether bonds and phenolic hydroxyl groups in different numbers, was found to be effective on *C. albicans* in either planktonic or biofilms states [17], [19], [20]. RCD also showed synergistic effect when used in combination with FLC against *C. albicans in vitro*, with the possible mechanism of interfering with the FLC-targeted ergosterol biosynthesis pathway [31]. Our understanding about the inhibition effect of RCD against *C. albicans* planktonic cells or biofilms is based on *in vitro* studies, while these experiments could not simulate the *in vivo* environment of the infection and host immune system exactly. In this study, we established an *in vivo* CVC-related *C. albicans* biofilms infective rabbit model to investigate the drug effect. We found that RCD has both prophylactic and therapeutic effect on *C. albicans* biofilms-associated infection by downregulating the expression of hypha specific genes. Also, when used in combination with traditional antifungal FLC, RCD could increase the efficacy against *C. albicans* biofilms. These findings would explain the efficacy and mechanisms of the anti-biofilms functions of RCD.

According to an *in vitro* study, *C. albicans* biofilms formation progresses in three development phases: early phase (0–11 h), intermediate phase (12–30 h) and mature phase (31–72 h) [32]. Based on the preliminary experiments, we found biofilms formation was related with strains and materials to which fungal cells adhered (data not shown). Using the CVC-related infective animal model, we obtained biofilms at different growth times of 10 h, 24 h, 48 h and 72 h. Observed with an inverted microscopy, the yeast cells and hypha were embeded in the dense extracellular matrix, with the structures not so clear as those formed *in vitro*. As to the 72-hour-old biofilms, we could not even distinguish yeast cells from the abundant extracellular matrix. The 24-hour-old biofilms yielded fungal cells of about 10^6^ CFU. The cell counts of biofilms changed little after 24 hours growth.

In the antifungal efficacy experiments, we administered the drug solutions to catheters locally by “antibiotic-lock” technique because it could form high concentration in the lumen locally to obtain higher antifungal effect than administered via veins. When injected in the early stage of biofilms development (4-hour-old), RCD was proved to have an inhibitory activity on *C. albicans* biofilms formed *in vivo* when used alone or in combination with FLC, indicating a prophylactic effect on biofilms-associated infection (Figure 3). When administered to the 24-hour-old biofilms for 5 days consecutively, RCD showed good anti-biofilms activity, especially in the medium or high dose groups. We could conclude from Figure 3 that RCD inhibited biofilms growth in a dose dependent style. Figure 5 indicated that RCD could inhibit the metabolic activity of fungal cells in biofilms. It has ever been demonstrated that RCD displayed a function in inhibiting biofilms formation by retarding fungal hypha formation [17]. In this *in vivo* experiment, although it is difficult to distinguish and count hypha because of the dense extracellular matrix and remaining tissues of animals, we could draw a similar conclusion as the *in vitro* experiment on biofilms inhibition of RCD.

The yeast-to-hypha transition of *C. albicans* biofilms is regulated by various impact factors, including carbohydrates, amino acids, salts, pH changes, temperature increases, but no single environmental factor could be defined as uniquely significant in stimulating the morphological switch [33]. To investigate the mechanisms of inhibitory effect of RCD on *C. albicans* biofilms *in vivo*, we selected genes interested for study further, including *ALS1, ALS3, ECE1, EFG1*, *HWP1* and *CDC35*. Previous study has proved that hypha formation in *C. albicans* biofilms is stimulated by the Ras-cAMP-Efg signalling pathway. In this cAMP dependent pathway, the GTP-bound form of Ras is capable of stimulating adenylate cyclase encoded by the gene *CDC35*, resulting in the increase of cAMP level that promotes specific gene activation, including *EFG1*, which induce the expression of the hypha specific genes such as *ALS3* and *HWP1*, and then mediate the hypha formation [34]. In *C. albicans*, the agglutinin-like sequence (ALS) genes have been revealed to have close relationship with biofilms formation [35], [36]. It was proved that overexpression of *ALS3* leads to an increase in biofilms mass [37], while overexpression of *HWP1*, the necessary factor for normal biofilms formation *in vitro* and *in vivo* could effectively restores the ability of a bcr1D null strain to form biofilms on murine mucosal surfaces [38]–[40]. In this study, we found all the genes were downregulated after RCD treatment compared with the control, indicating RCD affects *C. albicans* biofilms formation by inhibiting Ras-cAMP-Efg signalling pathway.

Our findings confirm the conclusion drawn from the *in vitro* study about the inhibition effect of RCD against *C. albicans* biofilms by using an *in vivo* CVC-related infective animal model. That is, RCD has activity on biofilms development when used alone or combined with FLC, with the mechanism of inhibition of hypha formation. Therefore, it could be concluded that RCD is a new kind of antifungal agent with potential in clinical application. Further researches need to be carried out to clarify the precise correlation between the upstream regulation genes and hypha-specific proteins.
